# Peripheral blood lymphocyte/monocyte ratio at the time of first relapse predicts outcome for patients with relapsed or primary refractory diffuse large B-cell lymphoma

**DOI:** 10.1186/1471-2407-14-341

**Published:** 2014-05-19

**Authors:** Yan-Li Li, Kang-Sheng Gu, Yue-Yin Pan, Yang Jiao, Zhi-Min Zhai

**Affiliations:** 1 Department of Hematology, The Second Affiliated Hospital of Anhui Medical University, Hefei, Anhui 230601, People’s Republic of China; 2 Department of Oncology, The First Affiliated Hospital of Anhui Medical University, Hefei, Anhui 230022, People’s Republic of China

**Keywords:** Absolute lymphocyte count/absolute monocyte count ratio, Diffuse large B-cell lymphoma, Relapse, SaaIPI, Survival

## Abstract

**Background:**

Despite the use of modern immunochemotherapy regimens, a significant proportion of diffuse large B-cell lymphoma (DLBCL) patients will relapse. We proposed absolute lymphocyte count/absolute monocyte count ratio (ALC/AMC ratio) as a new prognostic factor in relapsed or primary refractory DLBCL.

**Methods:**

We retrospectively analyzed 163 patients who have been diagnosed with relapsed or primary refractory DLBCL. The overall survival (OS) and progression-free survival (PFS) were measured from the time of first relapse. The Cox proportional hazards model was used to evaluate ALC/AMC ratio as prognostic factors for OS and PFS.

**Results:**

On univariate and multivariate analysis performed with factors included in the saaIPI, early relapse, prior exposure to rituximab and autologous stem-cell transplantation (ASCT), the ALC/AMC ratio at the time of first relapse remained an independent predictor of PFS and OS (PFS: P < 0.001; OS: P < 0.001). Patients with lower ALC/AMC ratio (<2.0) had lower overall response rate, 1-year PFS and 2-year OS rate compared with those with higher ALC/AMC ratio (≥2.0). Moreover, the ALC/AMC ratio can provide additional prognostic information when superimposed on the saaIPI.

**Conclusions:**

Lower ALC/AMC ratio at the time of first relapse is a adverse prognostic factor for OS and PFS in relapsed or primary refractory DLBCL, and leads to the identification of high-risk patients otherwise classified as low/intermediate risk by the saaIPI alone.

## Background

Diffuse large B-cell lymphoma (DLBCL) is the most common, accounts for 25%-30% of all newly diagnosed cases of adult Non-Hodgkin lymphoma (NHL). It is an aggressive lymphoma, but is potentially curable [1]. Despite the improvements in overall survival of patients with DLBCL with the routine addition of rituximab therapy; approximately one-third of the patients will develop relapsed/refractory disease that remains a major cause of morbidity and mortality [2]. Salvage chemotherapy followed by high-dose therapy and autologous stem-cell transplantation (ASCT) is the standard treatment for chemosensitive relapsed DLBCL [3]. Various parameters that greatly affect the results of salvage treatment in patients who have experienced relapse have been reported. From the Collaborative Trial in Relapsed Aggressive Lymphoma (CORAL) study, early relapse less than 12 months after diagnosis, the International Prognostic Index at relapse (saaIPI) and prior exposure to rituximab were detected as the parameters that affected 3-year event-free survival (EFS), progression-free survival (PFS), and overall survival (OS) [4].

Lymphocytes have an important role in immune surveillance in NHL, a view supported by the observation that lymphopenia is an adverse prognostic factor in NHL of various subtypes, including DLBCL [5-7]. Monocytes, which are considered immunologically relevant and are regarded as a surrogate marker of the tumor microenvironment, were also recently reported to be a prognostic factor in DLBCL [8-11], follicular lymphoma (FL) [12,13], T-cell lymphoma [14], extranodal natural killer/T-cell lymphoma (ENKL) [15] and Hodgkin’s Lymphoma (HL) [16,17]. Absolute lymphocyte count/absolute monocyte count ratio (ALC/AMC ratio) at diagnosis, as a simple biomarker combining an estimate of host immune homeostasis and tumor microenvironment, was recently shown to be an independent prognostic indicator in HL [16,17] and DLBCL [10,11]. However, to our best knowledge, there is no data available on whether the ALC/AMC ratio at the time of first relapse predicts outcome in patients with relapsed/primary refractory DLBCL. We, therefore, assessed the prognostic significance of ALC/AMC ratio at the time of first relapse.

## Methods

### Ethics statement

This study was approved by the Institutional Review Board (IRB) of the first affiliated and the second affiliated hospital of Anhui medical university. Study was performed in accord with the principles of the Declaration of Helsinki. All patients agreed to use their medical records for research.

### Patients

Consecutive 253 patients with DLBCL who had the full information, were evaluated and treated with CHOP (cyclophosphamide, hydroxydaunorubicin, vincristine, prednisone) or R-CHOP (rituximab-cyclophosphamIde, hydroxydaunorubicin, vincristine, prednisone) every 3 weeks for 3 to 8 cycles as first-line therapy and followed up between the years 2001 and 2011 at the first affiliated hospital and the second hospital of Anhui medical university, and 163 patients of them who had been diagnosed with relapsed/primary refractory. The patients who achieved CR/uCR/PR after second-line salvage chemotherapy entered the follow-up or ASCT, and the patients with no response after second-line salvage chemotherapy entered the clinical trial or supportive care. Second-line salvage chemotherapy regimens were: DHAP/R-DHAP (dexamethasone, cytarabine, and cisplatin/rituximab, dexamethasone, cytarabine, and cisplatin); DICE/R-DICE (dexamethasone, ifosfamide, cisplatin, and etoposide/rituximab, dexamethasone, ifosfamide, cisplatin, and etoposide); ICE/R-ICE (ifosfamide, carboplatin, and etoposide/rituximab, ifosfamide, carboplatin, and etoposide); GDP/R-GDP (gemcitabine, cisplatin, and dexamethasone/rituximab, gemcitabine, cisplatin, and dexamethasone). HIV-positive patients were excluded from this study.

### Study objective

The absolute lymphocyte count (ALC) and monocyte count (AMC) at the time of first relapse which were obtained from routine automated complete blood count (CBC); The absolute monocyte count/absolute lymphocyte count ratio (ALC/AMC ratio) was calculated by dividing the ALC by the AMC. Response criteria were based on the criteria from the International Harmonization Project [18], and evaluated after the third salvage chemotherapy course. Complete remission (CR) was defined by the disappearance of all documented disease; unconfirmed CR (CRu) was used when a residual mass was present without evidence of active disease. Partial response (PR) was defined as a 50% reduction of measurable disease. The primary endpoints were OS and PFS, defined as the time from the time of first relapse until last follow-up or death, and as the time from the time of first relapse to disease progression, relapse, or death of any cause or the last date of follow-up, respectively. Patient and disease characteristics included in the second-line IPI (sIPI) at the time of relapse or primary refractory disease [age < 60 vs. ≥ 60 years, Ann Arbor stage (III/IV vs. I/II), Karnofsky performance status (KPS) (<80% vs. ≥ 80%), lactate dehydrogenase (LDH) (normal vs. > normal) and number of extra nodal sites (ENS) involved (≤ vs. > 1)] were utilized.

### Statistical analysis

The correlation between the ALC, AMC, ALC/AMC ratio and clinical parameters was assessed by the chi-square test or Fisher’s exact test. PFS and OS were estimated using the Kaplan-Meier method and two-tailed log-rank test. Receiver operating characteristics analysis was also performed to determine the optimal cut-point for the ALC, AMC and ALC/AMC ratio. The Cox proportional hazards model was used to evaluate the ALC, AMC and ALC/AMC ratio as prognostic factors for PFS and OS and to adjust for other known prognostic variables included in the sIPI. P-values were not adjusted for multiple comparisons, All two-sided P-values < 0.05 were determined to be statistically significant. Statistical analysis was carried out using SPSS 16.0 software.

## Results

### Patient characteristics

We retrospectively analyzed data from a total of 253 DLBCL patients in this study, median follow-up following diagnosis was 36 months for the entire cohort (range: 3 month to 118 months) and the estimated 5 year OS for the entire cohort was 56%. Among 163 patients with evidence of first relapse, 42% had relapsed disease and 58% had primary refractory disease. The distribution of baseline characteristics for 163 relapsed/primary refractory patients based on an ALC/AMC ratio ≥ 2.0 versus ALC/AMC ratio < 2.0 at the time of first relapse is presented in Table 1. Eleven, Forty-four, sixty-four and forty-four patients treated with DHAP/R-DHAP, DICE/R-DICE, ICE/R-ICE, and GDP/R-GDP regimens, respectively, there was no significant difference in characteristic based on ALC/AMC ratio at the time of first relapse among the different second-line salvage chemotherapy (Table 1).

**Table 1:** Baseline characteristics based on relapsed/primary refractory DLBCL patients with an ALC/AMC ratio ≥ 2.0 versus ALC/AMC ratio < 2.0

**Characteristics**	**ALC/AMC ratio ≥ 2.0**	**ALC/AMC ratio < 2.0**	**P**
Disease status			
Primary reractory	37	57	<0.001
Relapse	53	16	
Age (years)			
<60	56	49	0.516
≥60	34	24	
Gender			
Male	46	27	0.096
Female	45	45	
Karnofsky			
Performance status			
80% or more	79	52	0.008
Less than 80%	11	21	
Number of extra nodal sites			
≤1	74	58	0.654
>1	16	15	
Ann Arbor Stage			
I/II	44	18	0.002
III/IV	16	55	
LDH			
≤Normal	65	40	0.021
>Normal	25	33	
SaaIPI			
0	34	13	0.002
1	34	23	
2	18	25	
3	4	12	
Initial chemotherapy			
CHOP	52	31	0.052
R-CHOP	38	42	
Rituximab-containing salvage therapy			
No	61	59	0.060
Yes	29	14	
ASCT			
No	78	67	0.300
Yes	12	6	
Salvage therapy			
DHAP	6	2	0.304
DICE	14	13	
ICE	23	25	
GDP	16	19	
R-DHAP	2	1	
R-DICE	10	7	
R-ICE	12	4	
R-GDP	7	2	

*Abbreviations:ALC/AMC ratio* absolute lymphocyte count/absolute monocyte count ratio, *LDH* lactate dehydrogenase, *saaIPI* second-line age-adjusted International Prognostic Index, *CHOP* cyclophosphamide, hydroxydaunorubicin, vincristine, prednisone, *R-CHOP* rituximab- cyclophosphamIde, hydroxydaunorubicin, vincristine, prednisone, *ASCT* autologous stem cell transplantation, *DHAP* dexamethasone, cytarabine, and cisplatin, *DICE* dexamethasone, ifosfamide, cisplatin, and etoposide, *ICE* ifosfamide, carboplatin, and etoposide, *GDP* gemcitabine, cisplatin, and dexamethasone, *R-DHAP* rituximab, dexamethasone, cytarabine, and cisplatin, *R-DICE* rituximab, dexamethasone, ifosfamide, cisplatin, and etoposide, *R-ICE* rituximab, ifosfamide, carboplatin, and etoposide, *R-GDP* rituximab, gemcitabine, cisplatin, and dexamethasone.

The ALC and AMC at the time of first relapse were derived from CBC counts. The cutoff points of ALC, AMC and ALC/AMC ratio for survival outcomes were selected by the receiver operating characteristic (ROC) curve analysis. The most discriminative cutoff value of ALC, AMC and ALC/AMC ratio was 1120/ul (area under the curve [AUC]: 0.648, 95% confidence interval: 0.563-0.733, P = 0.001), 530/ul (AUC: 0.734, 95% confidence interval: 0.658-0.811, P < 0.001) and 2.0 (AUC: 0.808, 95% confidence interval: 0.741-0.875, P < 0.001), respectively. In addition, The ALC and AMC at diagnosis were derived from pre-treatment CBC counts, and the cutoff points of ALC (1430/ul), AMC (460/ul) and ALC/AMC ratio (3.8) for survival outcomes were also selected by ROC curve analysis [11].

### Lower ALC/AMC ratio at the time of first relapse is a adverse prognostic factor for overall survival and progression free survival of relapsed/primary refractory DLBCL patients after second-line therapy

When the components of the sIPI (age ≥ 60 years; KPS < 80%; LDH > normal; Extranodal sites > 1; Ann Arbor stage III/IV) were assessed in univariate analysis by log rank, age was not predictive of PFS and OS (PFS: P = 0.531; OS: P = 0.693), whereas Extranodal sites (PFS: P = 0.054; OS: P = 0.029), KPS (P < 0.001 for both), LDH (P < 0.001 for both), and Ann Arbor stage (P < 0.001 for both) predicted PFS or OS. When entered into a Cox regression model for multivariate analysis, three factors, KPS, LDH, and Ann Arbor stage remain predictive (Additional file 1: Table S3 and Additional file 2: Table S4). These significant components were identical to those in the saaIPI, which was subsequently used to stratify patients into risk groups.

To determine the prognostic significance of the ALC, AMC and ALC/AMC ratio at the time of first relapse for OS and PFS of relapsed/primary refractory DLBCL patients, on univariate analysis, a relative reduction of ALC (<1120/ul), elevated AMC (≥530/ul) and lower ALC/AMC ratio (<2.0) associated with inferior OS (hazard ratio: 3.060, 95% confidence Interval: 1.878-4.988, P < 0.001; hazard ratio: 3.346, 95% confidence Interval: 2.178-5.141, P < 0.001; hazard ratio: 9.482, 95% confidence Interval: 5.497-16.355, P < 0.001; respectively). For comparison, ALC, AMC, ALC/AMC ratio at diagnosis, each of the three factors that comprise the saaIPI, early relapse (time from diagnosis to relapse of less than 12 months), prior rituximab treatment and ASCT or not was included in the analysis. Of these, ALC (<1430/ul), AMC (≥460/ul), ALC/AMC ratio (<3.8) and LDH (>normal) at diagnosis, LDH (>normal) at the time of first relapse, KPS (<80%), Ann Arbor stage (stage III/IV), time to relapse after diagnosis, months < 12 and ASCT or not were also of prognostic significance on univariate analysis (Table 2).

**Table 2:** Univariate and multivariate analyses for overall survival

**Prognostic factors**	**Univariate analysis**		**Multivariate analysis**	
	**HR (95% CI)**	**P**	**HR (95% CI)**	**P**
AMC ≥ 530/ul	3.346 (2.178-5.141)	<0.001	1.013 (0.537-1.913)	0.976
ALC < 1120/ul	3.060 (1.878-4.988)	<0.001	1.248 (0.704-2.211)	0.448
ALC/AMC ratio < 2.0	9.482 (5.497-16.355)	<0.001	8.758 (3.917-19.581)	<0.001
LDH > normal	2.440 (1.603-3.714)	<0.001	1.092 (0.643-1.855)	0.744
KPS < 80%	2.941 (1.852-4.670)	<0.001	1.004 (0.592-1.702)	0.989
Ann Arbor stage III/IV	3.088 (1.908-4.997)	<0.001	1.061 (0.581-1.937)	0.848
ALC/AMC ratio < 3.8	5.626 (3.119-10.174)	<0.001	1.184 (0.471-2.980)	0.719
AMC ≥ 460/ul	2.864 (1.831-4.482)	<0.001	1.243 (0.696-2.220)	0.462
ALC < 1430/ul	3.017 (1.855-4.907)	<0.001	1.387 (0.765-2.513)	0.281
LDH (at diagnosis > normal)	3.061 (1.963-4.775)	<0.001	1.400 (0.818-2.398)	0.220
Time to relapse after diagnosis, months <12	7.003 (4.162-11.783)	<0.001	3.527 (1.597-7.787)	0.002
Prior rituximab treatment	0.616 (0.592-1.364)	0.899	-	-
Not ASCT	4.984 (1.819-13.655)	0.002	3.877 (1.310-11.476)	0.014

*Abbreviations:HR* hazard ratio, *CI* confidence Interval, *AMC* absolute monocyte count, *ALC* absolute lymphocyte count, *ALC/AMC ratio* absolute lymphocyte count/absolute monocyte count ratio, *LDH* lactate dehydrogenase, *KPS* Karnofsky Performance status, *ASCT* autologous stem cell transplantation.

Then we included components of the saaIPI in a multivariate analysis with the ALC, AMC, ALC/AMC ratio at diagnosis and at the time of first relapse, time to relapse after diagnosis, months < 12 and ASCT or not. As summarized in Table 2, the ALC/AMC ratio at the time of first relapse, early relapse (time to relapse after diagnosis, months < 12) and ASCT or not were independently significant prognostic factors for OS, with hazard ratios of 8.758 (95% confidence Interval: 3.917-19.581, P < 0.001), 3.527 (95% confidence Interval: 1.597-7.787, P = 0.002) and 3.877 (95% confidence Interval: 1.310-11.476, P = 0.014), respectively (Table 2). Similarly, the ALC/AMC ratio at the time of first relapse, early relapse (time to relapse after diagnosis, months < 12) and ASCT or not were independently significant predictors of PFS when adjusted for components of the saaIPI on multivariate analysis (Additional file 3: Table S5).

### Response and survival rate according to prognostic factors

After platinum-based second-line salvage chemotherapy, the overall response rate, including CR, CRu and PR, was 49%. The factors significantly affecting the overall response rate included early relapse (time to relapse after diagnosis, months < 12), saaIPI of 2 to 3, prior rituximab treatment, ALC/AMC ratio and LDH at the time of first relapse (P < 0.001) (Table 3). After a median follow-up time of 13 months, the 1-year PFS rate was 37% and was significantly different between the ALC/AMC ratio < 2.0 and ALC/AMC ratio ≥ 2.0 (12% and 58%, respectively; P < 0.001). 2-year OS was 26%, with significant difference between the ALC/AMC ratio < 2.0 and ALC/AMC ratio ≥ 2.0 (4% and 43%, respectively; P < 0.001). 1-year PFS and 2-year OS were also affected by early relapse (time to relapse after diagnosis, months < 12), saaIPI, rituximab-containing salvage therapy, the ALC, AMC and LDH at the time of first relapse (Table 3).

**Table 3:** Response rate and survival according to prognostic factors

**Characteristic**	**Response CR/CRu/PR**			**1-year progression free survival**			**2-year overall survival**		
	**N**	**%**	**P**	**N**	**%**	**P**	**N**	**%**	**P**
Time to relapse after diagnosis, months									
<12	33	35	<0.001	15	16	<0.001	7	7	<0.001
≥12	47	68		46	67		35	51	
Prior rituximab treatment									
No	63	67	<0.001	38	40	0.355	20	21	0.126
Yes	17	25		23	33		22	32	
SaaIPI at relapse									
2–3	16	27	<0.001	10	17	<0.001	5	9	<0.001
0–1	64	67		51	49		37	36	
ALC/AMC ratio									
<2.0	20	27	<0.001	9	12	<0.001	3	4	<0.001
≥2.0	60	67		52	58		39	43	
Absolute monocyte count									
≥530/ul	21	33	<0.001	13	20	<0.001	6	9	<0.001
<530/ul	59	60		48	49		36	36	
Absolute lymphocyte count									
<1120/ul	41	41	0.015	27	27	0.001	15	15	<0.001
≥1120/ul	39	61		34	53		27	42	
LDH at relapse									
>Normal	16	28	<0.001	12	21	0.001	9	16	0.026
≤Normal	64	61		49	47		33	31	
Rituximab containing salvage therapy									
No	55	46	0.166	39	33	0.036	22	18	<0.001
Yes	25	58		22	51		20	47	

*Abbreviations:CR* complete remission, *CRu* unconfirmed complete remission, *PR* partial response, *saaIPI* second-line age-adjusted International Prognostic Index, *ALC/AMC ratio* absolute lymphocyte count/absolute monocyte count ratio, *LDH* lactate dehydrogenase.

### The ALC/AMC ratio at the time of first relapse and second-line therapy

The ALC/AMC ratio at the time of first relapse was analyzed to determine whether it could further discriminate for survival when considering second-line therapy with either ASCT or further chemotherapy. In the 18 first relapsed DLBCL treated with ASCT, the median OS and PFS were significantly longer for patients with an ALC/AMC ratio ≥ 2.0 when compared with those patients with an ALC/AMC ratio < 2.0 (median OS: 34 months, 2 years OS rates of 92% versus median OS: 19 months, 2 years OS rates of 17%, P = 0.001; and median PFS: 27 months,1 years PFS rates of 92% versus median PFS: 15 months, 1 years PFS rates of 83%, P = 0.596, respectively). In the 145 first relapsed DLBCL patients that were treated with further chemotherapy, the median OS and PFS were also significantly longer for patients with an ALC/AMC ratio ≥ 2.0 when compared with those patients with an ALC/AMC ratio < 2.0 (median OS: 18 months, 2 years OS rates of 36% versus median OS: 8 months, 2 years OS rates of 3%, P < 0.001; and median PFS: 12 months, 1 years PFS rates of 53% versus median PFS: 5 months, 1 years PFS rates of 6%, P < 0.001, respectively).

### The ALC/AMC ratio at the time of first relapse identifies high-risk patients and provides additional prognostic information when superimposed on the saaIPI

**Figure 1:**
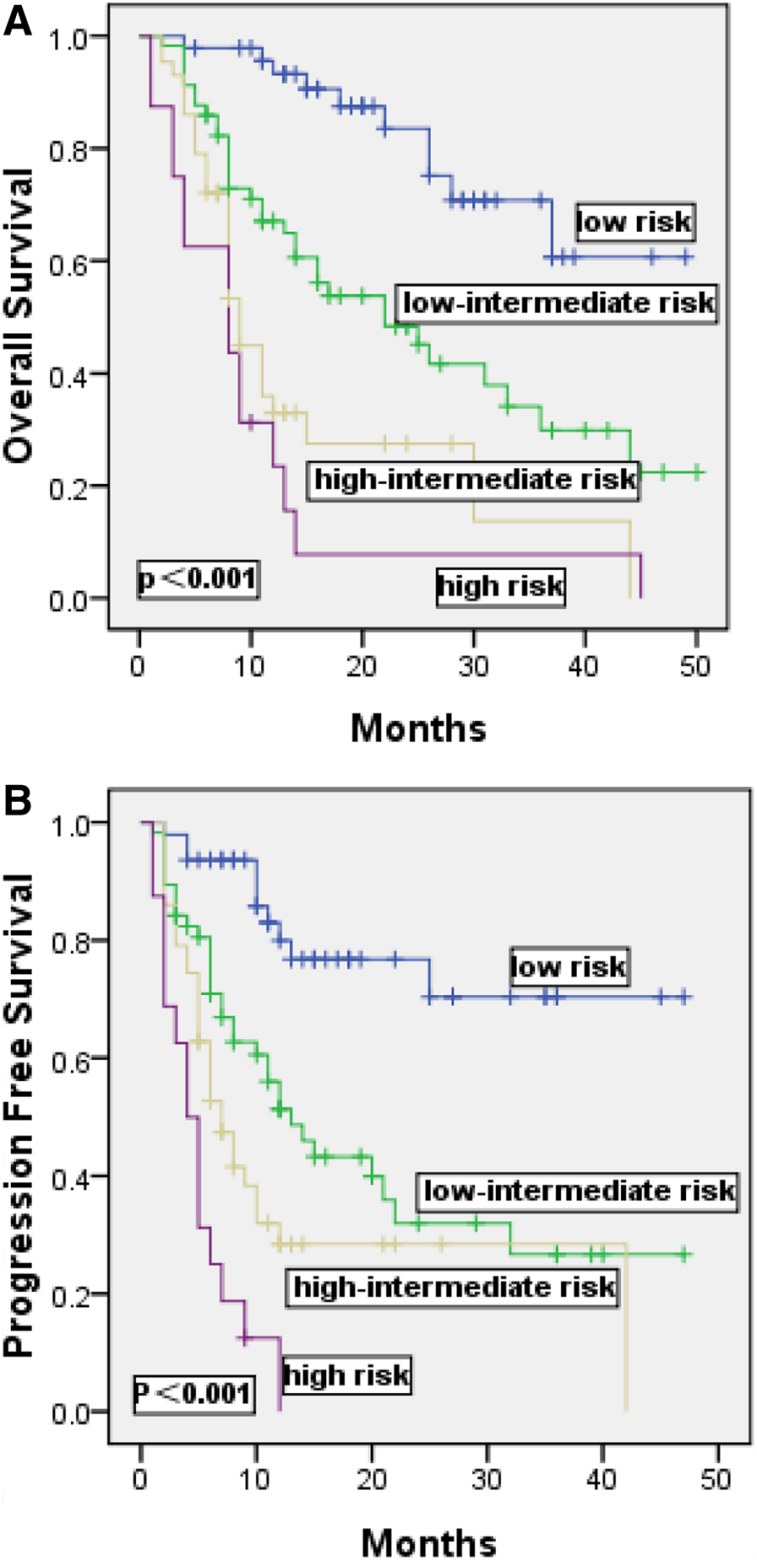
Kaplan-Meier estimates of overall survival (A) and progression-free survival (B) for the 163 relapsed/primary refractory DLBCL patients stratified by second-line age-adjusted International Prognostic Index (saaIPI) are shown.

**Figure 2:**
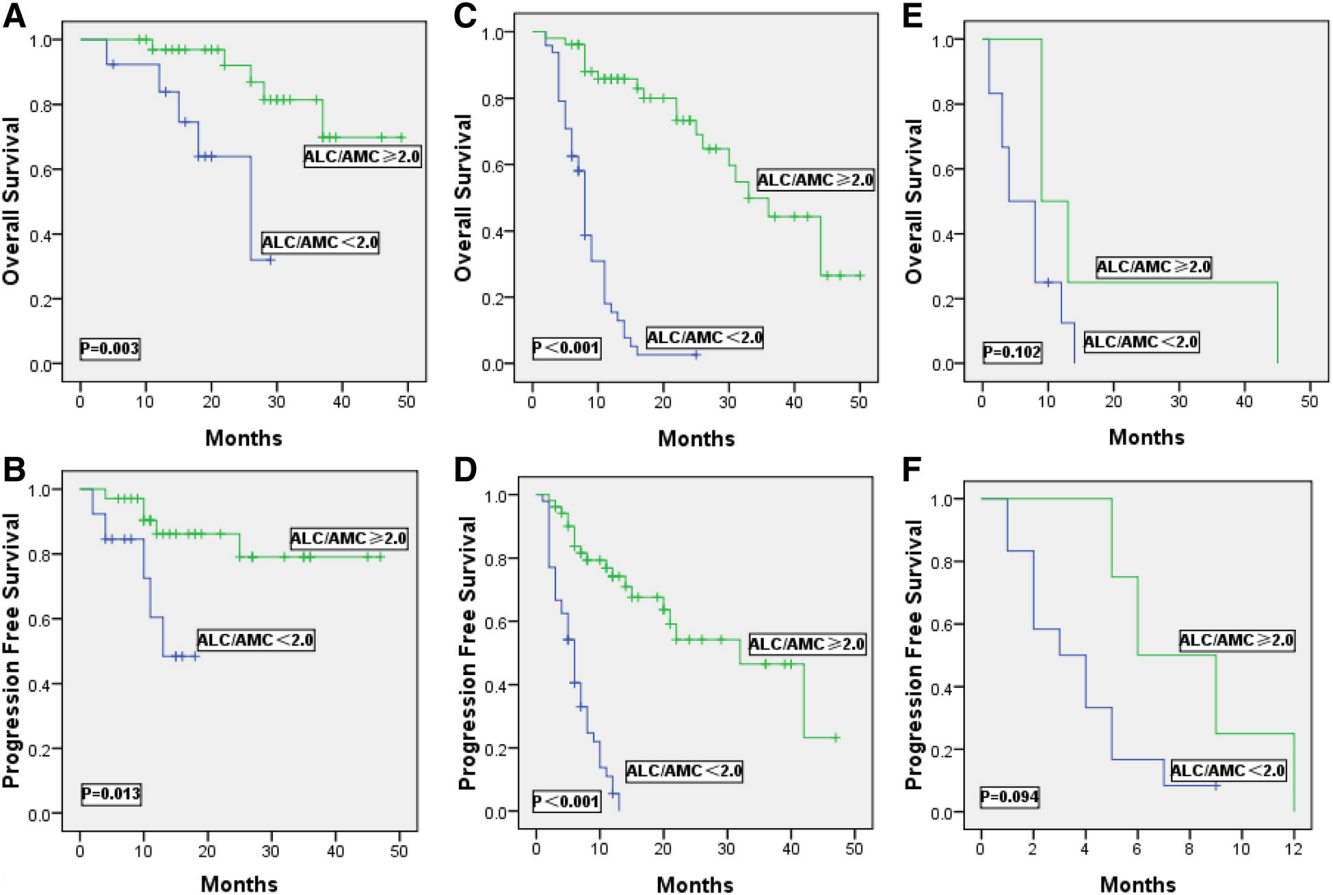
Kaplan-Meier estimates of overall survival (A, C, E) and progression- free survival (B, D, F) for the 163 relapsed/primary refractory DLBCL patients stratified by the saaIPI as either low- (A, B), low-intermediate/high-intermediate (C, D) or high risk (E, F) were further stratified into low or high groups by the ALC/AMC ratio.

PFS and OS were analyzed using the saaIPI in Figure 1A and B. The ALC/AMC ratio at the time of first relapse remains an independently significant prognostic factor when adjusting for the saaIPI. Therefore, we sought to determine whether it may provide additional prognostic information when combined with the saaIPI. The 47 low-risk, 100 intermediate-risk (high-intermediate and low-intermediate were combined) and 16 high-risk patients identified by the saaIPI were subsequently risk stratified using the ALC/AMC ratio. We showed that patients with a low-risk category of saaIPI score (saaIPI = 0) and low-intermediate/high-intermediate (saaIPI = 1–2), the ALC/AMC ratio was a useful way to distinguish those with favorable outcomes from those with adverseand outcomes (OS: P = 0.003, PFS: P = 0.013, Figure 2A and B; OS: P < 0.001, PFS: P < 0.001, Figure 2C and D; respectively), in conclusion, the ALC/AMC ratio was able to further risk-stratify these patients. But in patients with high-risk (saaIPI = 3), the number of the patients were only sixteen, so as likely not to make a similar analysis in this subgroup meaningful (OS: P = 0.102, PFS: P = 0.094, Figure 2E and F). In addition, we analysed 94 primary refractory and 69 relapsed DLBCL patients to seek to determine whether it may provide additional prognostic information when combined with the saaIPI, respectively. We showed that in primary refractory and relapsed patients with a low-intermediate/high-intermediate (saaIPI = 1–2), respectively, the ALC/AMC ratio was a useful way to distinguish those with favorable outcomes from those with adverse outcomes (OS: P < 0.001, PFS: P < 0.001, Additional file 4: Figure S1C and D; OS: P < 0.001, PFS: P < 0.001, Additional file 5: Figure S2C and D; respectively).

**Figure 3:**
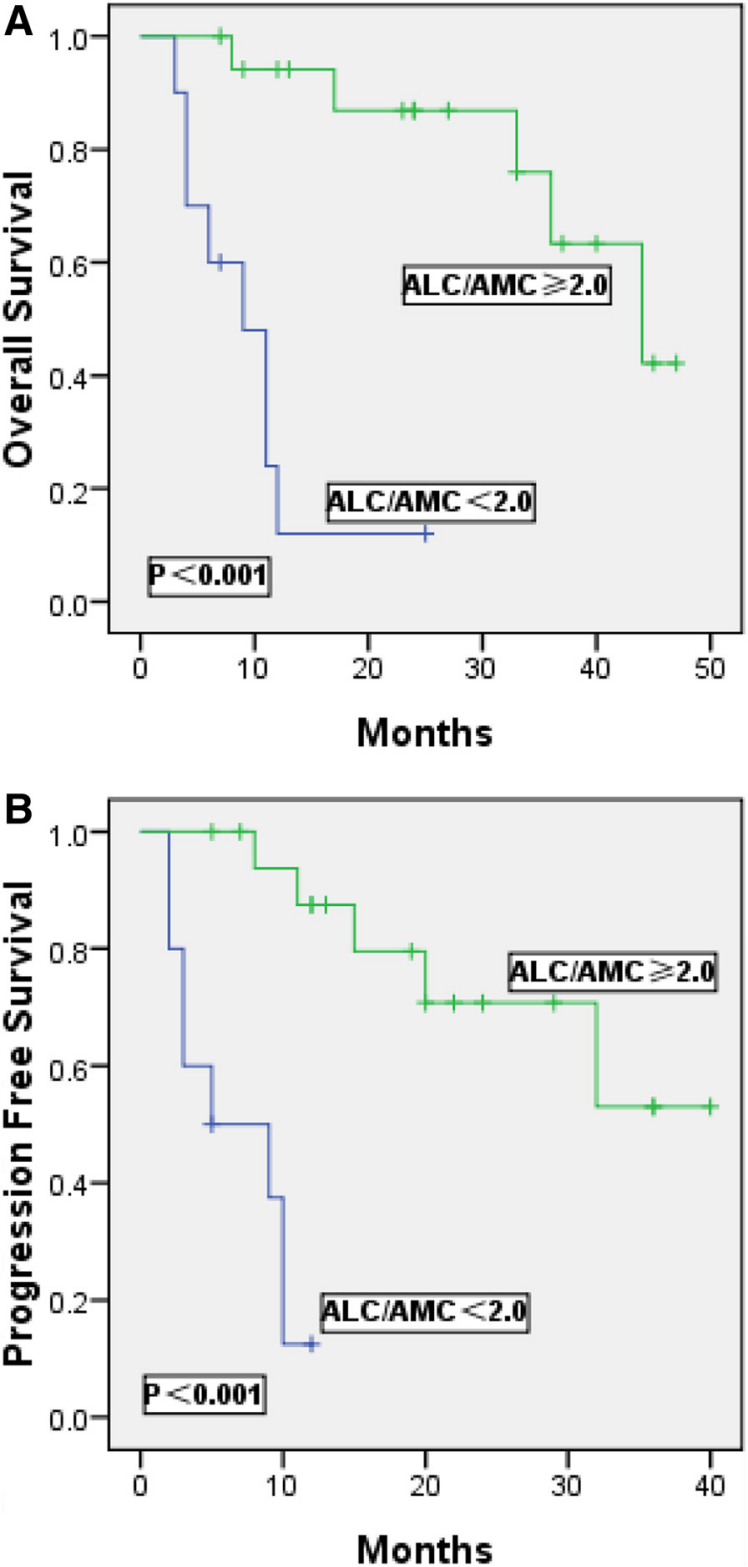
Kaplan-Meier estimates of overall survival (A) and progression-free survival (B) for 43 relapsed/primary refractory DLBCL patients treated with rituximab-salvage therapy identified by the saaIPI as intermediate risk (saaIPI =1-2) were further stratified into low or high groups by the ALC/AMC ratio.

Among the all patients, 8% of patients identified by the saaIPI as ‘low -risk’, upon further risk stratification by the ALC/AMC ratio (<2.0) at the time of first relapse, found to have dismal outcomes, with a median OS of 18 months, a median PFS of 10 months; Moreover, 29% of identified as ‘intermediate -risk’ patients were with a median OS of 8 months, a median PFS of 6 months. Similar results were obtained when intermediate risk patients treated with rituximab-containing salvage therapy were risk-stratified by the ALC/AMC ratio in Figure 3. In this case, 26% of identified as ‘intermediate risk’ patients were with a median OS of 15 months, a median PFS of 12 months.

## Discussion

The International Prognostic Index (IPI), solely considering patient and tumor characteristics, is currently the standard prognostic tool used to predict clinical outcomes for patients with DLBCL. But recent work, based on gene expression profiling studies in NHL, shows that gene expression by tumor-infiltrating lymphocytes and myeloid-derived cells predict a clinical outcome [19], which implies that a prognostic system that considers features of the tumor- bearing host and the tumor microenvironment may provide prognostic information. The ALC/AMC ratio at diagnosis, as a simple biomarker combining an estimate of host immune homeostasis and tumor microenvironment, was recently shown to be an independent prognostic indicator in HL [16,17] and DLBCL [10], and combining the dichotomized ALC and AMC to generate the ALC/AMC prognostic score was also provided prognostic information independently of that included in the IPI [8,9]. Therefore, we first sought to examine the both in our 253 DLBCL patients from the two hospital institution. In our study [11], the results were consistent with the previous findings from Wilcox RA et al., Batty N et al. and Rambaldi A et al. [8-10].

The international Prognostic Index at relapse (saaIPI), early relapse less than 12 months after diagnosis and prior exposure to rituximab have been demonstrated to be predictors of clinical outcomes in first relapsed DLBCL patients [4,20]. Biologically, a few parameters at relapse have been reported to be predictive of survival independent of IPI score, including ALC at the time of first relapse [21]. No reports have addressed whether ALC/AMC ratio at the time of first relapse predicts survival in NHL. Thus, we assessed the prognostic significance of ALC/AMC ratio at the time of first relapse in relapsed/primary refractory DLBCL. The present study showed that ALC/AMC ratio at the time of first relapse was a adverse independent prognostic factor for OS and PFS and can identify the high-risk patients otherwise classified as low/intermediate risk by the saaIPI alone. We also found that ALC/AMC ratio at the time of first relapse and several independent factors significantly affected response rates after salvage therapy, including saaIPI score, early relapse less than 12 months after diagnosis, and prior rituximab treatment, which is in agreement with those provided by Gisselbrecht C et al. [4]. ALC/AMC ratio at the time of first relapse, early relapse less than 12 months after diagnosis and saaIPI score, the same independent factors were found for 1-year PFS and 2-year OS rate. But there were no difference between the prior rituximab treatment or not; the fact that most patients in our study who progressed after R-CHOP have late relapse may explain this discrepancy. In accordance with previous reports [22,23], in our study, those who received rituximab-containing salvage therapy at relapse achieved significantly longer survival (both PFS and OS) and had a significant improvement in the 1-year PFS and 2-year OS rates than those who underwent salvage therapy with chemotherapy alone regardless of the first-line treatment with CHOP or R-CHOP, and ASCT or not. In addition, among patients who received first-line treatment with CHOP, those who received rituximab-containing salvage therapy at relapse achieved significantly improvement in 2-year OS rate than those who underwent salvage therapy with chemotherapy alone.

lymphopenia is considered a surrogate marker of host immunological incompetence, in addition, lymphocytes (including natural killer [NK] cells) are important mediators of antibody-dependent cell-mediated cytotoxicity, and may be required for rituximab-mediated, antibody-dependent cellmediated cytotoxicity-dependent destruction of malignant B cells [24]. Not surprisingly then, lymphopenia is an adverse prognostic factor in indolent and aggressive NHL, including DLBCL. Recently, Dehghani M et al. [25] and Gergely L et al. [26] reported that lower CD4+ lymphocyte, CD3+ and CD8+ lymphocytes were corresponding with significantly inferior overall survival in B-cell NHL, respectively. Głowala-Kosińska M et al. [27] showed that lower number of circulating regulatory T cells (Tregs) was associated with reduced chance of achieving CR and reduced probability of even-free survival (EFS) in newly diagnosed DLBCL, and Shafer D et al. [28] showed that low NK cell counts in peripheral blood were associated with inferior overall survival in patients with FL. However, in the tumor microenvironment, elevated infiltration of FOXP3+ Tregs was correlated with a favorable clinical outcome in different types of lymphoma reported, including DLBCL [29-33], and Hasselblom S et al. reported that DLBCL patients with a small number of cytotoxic T-cell intracytoplasmic antigen-1 (TIA-1) + T cellshad significantly better outcome [34].

Myeloid-lineage cells, including monocytes and their progeny, promote tumorigenesis and angiogenesis [35], and contribute to the suppression of host antitumor immunity so that not surprisingly then, development of peripheral blood neutrophilia or monocytosis are adverse prognostic factors in multiple solid tumors [36-38]. A new nomenclature defines human monocyte subsets into three, classical (CD14++CD16-), intermediate (CD14++CD16+) and nonclassical (CD14 + CD16++) [39]. CD16+ monocytes recently have shown diagnostic and prognostic potential in malignant disease [40-42]. but to date, as far as we know, are not investigated in lymphoma. Monocytes that circulate in the bloodstream are recruited to inflamed tissues and give rise to macrophages. Macrophages, which termed tumour-associated macrophages (TAMs), play an important role in tumor tissues. TAMs can be classified into two functionally distinct types, M1 and M2, which reported to determine the effects against tumors, i.e. promotional (M2) or suppressive (M1) [43]. Hasselblom S et al. [44] reported that the number of TAMs in DLBCL tissues was not correlated with the prognosis, but the recent study of the Osaka Lymphoma Study Group [45] showed that a high number of M2 TAMs, but not of total TAMs, was an independent factor for a significantly poor prognosis in DLBCL patients.

The pattern of human monocytes recruitment in vivo to tumors is not very clear, although Qian et al. [46] recently showed that human CD14 + CD16- inflammatory monocytes recruited by a CCL2 mechanism and differentiate into macrophages that promote the subsequent growth of metastatic cells in vivo. In addition, Nakasone ES et al. [47] reported that infiltration of CCR2- expressing myeloid cells into chemotherapy-treated tumors contributes to tumor regrowth and relapse after treatment; recently, Sanford DE et al. [48] found that inflammatory monocyte (CD14+/CCR2+) recruitment is critical to pancreatic cancer progression, and targeting CCR2 may be an effective immunotherapeutic strategy in this disease. As previously mentioned, We hypothesized that several important questions could remain: How do the subsets of blood monocyte exist in DLBCL patients at the time of first relapse? Do the monocyte subsets vary when compared with the time of diagnosis? Which blood monocyte subsets preferentially recruit to metastatic sites, and involve in tumor microenvironment? Are there novel specific therapeutic strategies to inhibit the monocyte recruitment, which may promote the metastasis and resistance to chemotherapy, so that can increase the response rate of second-line salvage regimens, prolong the overall survival? Thus, more further studies are deserved. In a word, the novel therapy of relapsed DLBCL resulting from better understanding of patient, tumor characteristics, host immunity and tumor microenvironment may be needed.

Our study has some limitations. First, although there was no significant difference in characteristic based on ALC/AMC ratio at the time of first relapse and response rate among different second-line salvage chemotherapy in our study, and no clear superiority of one salvage regimen over another has been demonstrated all over the world, this may lower the quality of the data. Thus, further studies exploring the prognostic significance of ALC/AMC ratio at the time of first relapse in relapsed/primary refractory DLBCL patients with uniform salvage regimens are warranted. Second, on the one hand, as a retrospective study, patients were not randomly assigned to ASCT versus other selvage therapies, which meant that the choice of ASCT or not might have been biased by the treating physician’s preference based on patient’s characteristics. Thus, even though ASCT was found to be a prognostic factor for survival in our study, it is important to reemphasize the potential bias in patient selectivity undergoing ASCT; on the other hand, the number of patients who received ASCT was small in our study, so this should require validation in a larger cohort in the future. However, our study reported a significantly superior OS and PFS in patients who underwent ASCT compared with who received further chemotherapy, which was in agreement with the result of the 1995 PARMA trial [49]. The patients with higher ALC/AMC ratio experienced better OS and PFS regardless of their treatment (ASCT or not), and the ALC/AMC ratio at the time of first relapse was able to discriminate for survival in both groups (ASCT and further chemotherapy).

## Conclusions

In conclusion, our study identifies prognostic utility for ALC/AMC ratio at the time of first relapse as a simple tool in relapsed/primary refractory DLBCL patients. Given the limited number of patients included in this retrospective study, the prognostic value will require validation in an independent cohort of patients in prospective trials, especially in chemosensitive relapsed DLBCL followed by high-dose therapy and stem cell transplantation. To our our knowledge, this study is the first to identify ALC/AMC ratio the prognostic significance independent of the saaIPI to predict response rate and survival outcome in relapsed/refractory DLBCL patients and add to its ability to identify high-risk patients. As new immuno-based therapies are developed to treat relapsed NHL, the role of the host immune homeostasis and tumor microenvironment, such as targeting monocyte mobilization, is becoming more important in these treatment modalities.

## Competing interests

The authors declared that they have no competing interests.

## Authors’ contributions

YLL designed the study, erformed the statistical analysis, and drafted the manuscript. KSG, YYP and YJ participated in the collection of the clinical data. ZMZ conceived of the study, and participated in its design and coordination and helped to draft the manuscript. All authors read and approved the final manuscript.

## Pre-publication history

The pre-publication history for this paper can be accessed here:

http://www.biomedcentral.com/1471-2407/14/341/prepub

## Supplementary Material

Additional file 1 sIPI as predictors of overall survival. Click here for file

Additional file 2 sIPI as predictors of progression free survival. Click here for file

Additional file 3 Univariate and multivariate analyses for progression free survival. Click here for file

Additional file 4 Kaplan-Meier estimates of overall survival (A, C, E) and progression-free survival (B, D, F) for 94 primary refractory DLBCL patients identified by the saaIPI as either low- (A, B), low-intermediate/high- intermediate (C, D) and high risk (E, F) were further stratified into low or high groups by the ALC/AMC ratio. Click here for file

Additional file 5 Kaplan-Meier estimates of overall survival (A, C) and progression-free survival (B, D) for 69 relapsed DLBCL patients identified by the saaIPI as either low- (A, B), low-intermediate/high-intermediate (C, D) were further stratified into low or high groups by the ALC/AMC ratio. Click here for file
