# Supplementary material for: Peripheral blood lymphocyte/monocyte ratio at the time of first relapse predicts outcome for patients with relapsed or primary refractory diffuse large B-cell lymphoma
Source: BMC Cancer. 2014 May 19;14:341. doi: 10.1186/1471-2407-14-341 (PMC4033684; doi:10.1186/1471-2407-14-341)
Supplement: Additional file 1 — sIPI as predictors of overall survival. [file 1471-2407-14-341-S1.doc]

**Additional file 1:** sIPI as predictors of overall survival.

| **Prognostic factors** | | **Univariate analysis** | | |  | **Multivariate analysis** | | |  |
| --- | --- | --- | --- | --- | --- | --- | --- | --- | --- |
|  |  | **HR(95%CI)** | **P** | |  | **HR(95%CI)** | **P** | |  |
| Age≥60  KPS＜80%  Extranodal sites＞1  AnnArbor stageⅢ/Ⅳ  LDH＞normal |  | 1.090(0.711-1.671)  2.941(1.852-4.670)  1.745(1.057-2.879)  3.088(1.908-4.997)  2.440(1.603-3.714) | | 0.693  ＜0.001  0.029  ＜0.001  ＜0.001 | | 1.250(0.809-1.931)  1.910(1.161-3.140)  0.953(0.558-1.625)  2.446(1.436-4.166)  1.999(1.292-3.093) | | 0.315  0.011  0.859  0.001  0.002 | |
|  |  |  |  | |  |  | |  |  |

Abbreviations: HR, hazard ratio; CI, confidence Interval; KPS, Karnofsky Performance status; LDH, lactate dehydrogenase.
