# Supplementary material for: Peripheral blood lymphocyte/monocyte ratio at the time of first relapse predicts outcome for patients with relapsed or primary refractory diffuse large B-cell lymphoma
Source: BMC Cancer. 2014 May 19;14:341. doi: 10.1186/1471-2407-14-341 (PMC4033684; doi:10.1186/1471-2407-14-341)
Supplement: Additional file 2 — sIPI as predictors of progression free survival. [file 1471-2407-14-341-S2.doc]

**Additional file 2:** sIPI as predictors of progression free survival.

| **Prognostic factors** |  | **Univariate analysis** | | |  | | **Multivariate analysis** | | |  |
| --- | --- | --- | --- | --- | --- | --- | --- | --- | --- | --- |
|  |  | **HR(95%CI)** | **P** | | |  | **HR(95%CI)** | **P** | |  |
| Age≥60  KPS＜80%  Extranodal sites＞1  AnnArbor stageⅢ/Ⅳ  LDH＞normal |  | 1.146(0.749-1.751)  2.905(1.829-4.615)  1.636(0.993-2.698)  2.724(1.688-4.398)  2.642(1.726-4.043) | | 0.531  ＜0.001  0.054  ＜0.001  ＜0.001 | | | 1.210(0.785-1.866)  2.303(1.406-3.774)  0.839(0.488-1.444)  2.081(1.227-3.527)  2.267(1.459-3.522) | | 0.388  0.001  0.526  0.007  ＜0.001 | |
|  |  |  |  | |  | |  |  | |  |

Abbreviations: HR, hazard ratio; CI, confidence Interval; KPS, Karnofsky Performance status; LDH, lactate dehydrogenase.
