# Supplementary material for: Peripheral blood lymphocyte/monocyte ratio at the time of first relapse predicts outcome for patients with relapsed or primary refractory diffuse large B-cell lymphoma
Source: BMC Cancer. 2014 May 19;14:341. doi: 10.1186/1471-2407-14-341 (PMC4033684; doi:10.1186/1471-2407-14-341)
Supplement: Additional file 3 — Univariate and multivariate analyses for progression free survival. [file 1471-2407-14-341-S3.doc]

**Additional file 3:** Univariate and multivariate analyses for progression free survival.

| **Prognostic factors** |  | **Univariate analysis** | | |  | **Multivariate analysis** | | |  | |
| --- | --- | --- | --- | --- | --- | --- | --- | --- | --- | --- |
|  |  | **HR(95%CI)** | **P** | |  | **HR(95%CI)** | **P** | |  | |
| AMC≥530/ul |  | 3.196(2.076-4.919) | | ＜0.001  ＜0.001  ＜0.001  ＜0.001  ＜0.001  ＜0.001  ＜0.001  ＜0.001  ＜0.001  ＜0.001  ＜0.001  0.347  0.002 | | 1.002(0.527-1.903) | | 0.996 | |  |
| ALC＜1010/ul  ALC/AMC ratio＜2.0  LDH＞normal  KPS＜80%  AnnArbor stageⅢ/Ⅳ  ALC/AMC ratio＜3.8  AMC≥460/ul  ALC＜1430/ul  LDH(at diagnosis)＞normal  Time to relapse after diagnosis, months ＜12)  Prior rituximab treatment  Not ASCT |  | 2.927(1.801-4.756)  7.165(4.312-11.905)  2.642(1.726-4.043)  2.905(1.829-4.615)  2.724(1.688-4.398)  4.572(2.534-8.248)  2.355(1.511-3.671)  2.780(1.710-4.519)  2.377(1.542-3.663)  6.027(3.537-10.271)  0.818(0.539-1.243)  5.106(1.860-14.016 ) | |  |  | 1.648(0.945-2.872)  5.501(2.643-11.448)  1.609(0.917-2.823)  1.716(1.033-2.850)  1.008(0.575-1.769)  0.710(0.288-1.756)  1.315(0.750-2.305)  1.508(0.808-2.813)  0.835(0.486-1.434)  3.750(1.756-8.005)  -  5.076(1.682-15.322) | | 0.078  ＜0.001  0.097  0.037  0.977  0.459  0.339  0.197  0.513  0.001  -  0.004 | | |

Abbreviations: HR, hazard ratio; CI, confidence Interval; AMC, absolute monocyte count; ALC, absolute lymphocyte count; ALC/AMC ratio, absolute lymphocyte count/absolute monocyte count ratio; LDH, lactate dehydrogenase; KPS, Karnofsky Performance status; ASCT, autologous stem cell transplantation.
