# Supplementary material for: Peripheral blood lymphocyte/monocyte ratio at the time of first relapse predicts outcome for patients with relapsed or primary refractory diffuse large B-cell lymphoma
Source: BMC Cancer. 2014 May 19;14:341. doi: 10.1186/1471-2407-14-341 (PMC4033684; doi:10.1186/1471-2407-14-341)
Supplement: Additional file 4 — Kaplan-Meier estimates of overall survival (A, C, E) and progression-free survival (B, D, F) for 94 primary refractory DLBCL patients identified by the saaIPI as either low- (A, B), low-intermediate/high- intermediate (C, D) and high risk (E, F) were further stratified into low or high groups by the ALC/AMC ratio. [file 1471-2407-14-341-S4.doc]

**Additional file 4**


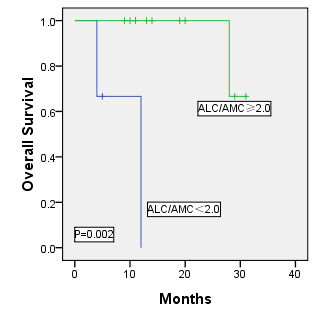
 **(A)**


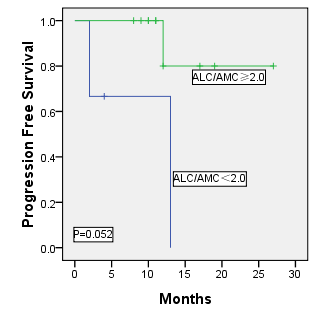
 **(B)**


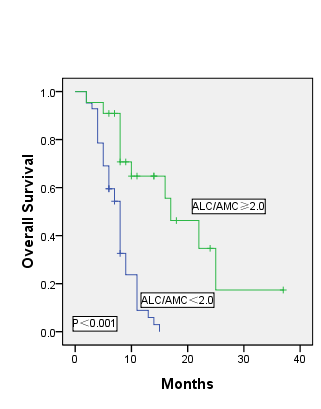
 **(C)**


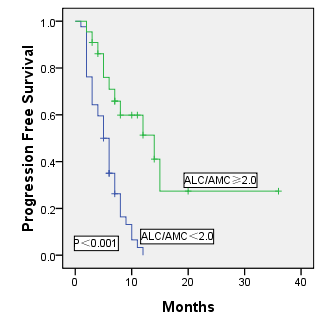
  **(D)**


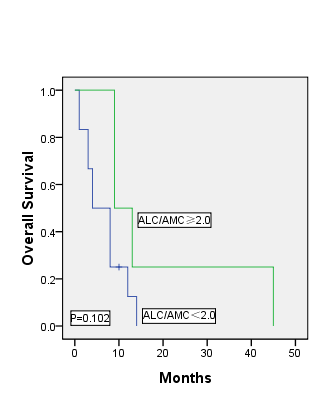
 **(E)**


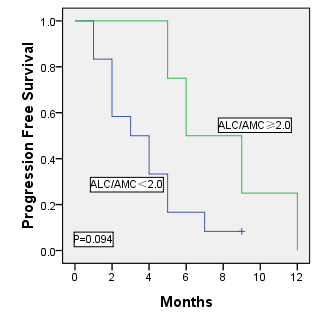
 **(F)**

**Additional file 4:** Kaplan-Meier estimates of overall survival (A,C,E) and progression-free survival (B,D,F) for 94 primary refractory DLBCL patients identified by the saaIPI as either low- (A,B), low-intermediate/high- intermediate (C,D) and high risk (E,F) were further stratified into low or high groups by the ALC/AMC ratio.
