# Supplementary material for: Peripheral blood lymphocyte/monocyte ratio at the time of first relapse predicts outcome for patients with relapsed or primary refractory diffuse large B-cell lymphoma
Source: BMC Cancer. 2014 May 19;14:341. doi: 10.1186/1471-2407-14-341 (PMC4033684; doi:10.1186/1471-2407-14-341)
Supplement: Additional file 5 — Kaplan-Meier estimates of overall survival (A, C) and progression-free survival (B, D) for 69 relapsed DLBCL patients identified by the saaIPI as either low- (A, B), low-intermediate/high-intermediate (C, D) were further stratified into low or high groups by the ALC/AMC ratio. [file 1471-2407-14-341-S5.doc]

**Additional file 5**


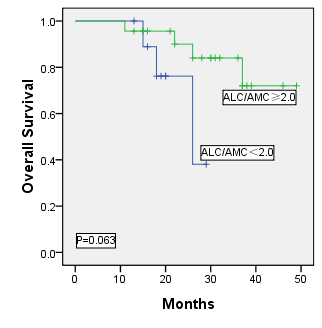
 **(A)**


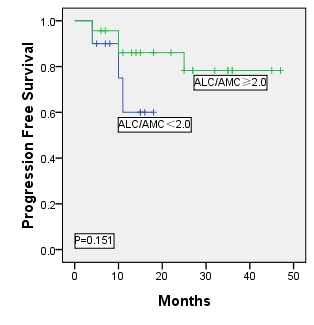
 **(B)**


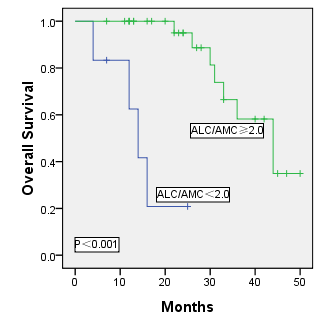
 **(C)**


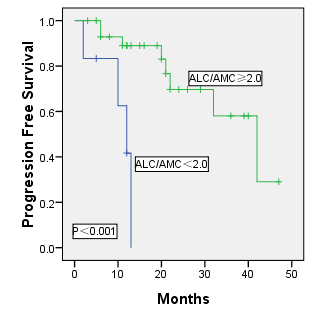
 **(D)**

**Additional file 5:** Kaplan-Meier estimates of overall survival (A,C) and progression-free survival (B,D) for 69 relapsed DLBCL patients identified by the saaIPI as either low- (A,B), low-intermediate/high-intermediate (C,D) were further stratified into low or high groups by the ALC/AMC ratio.
